# Downregulated miR-45 Inhibits the G1-S Phase Transition by Targeting Bmi-1 in Breast Cancer: Erratum

**DOI:** 10.1097/01.md.0000470923.58820.14

**Published:** 2015-08-07

In the article “Downregulated miR-45 Inhibits the G1-S Phase Transition by Targeting Bmi-1 in Breast Cancer”,^1^ which appeared in Volume 94, Issue 21 of *Medicine*, the title was presented incorrectly. The correct title should have read Downregulated miR-495 Inhibits the G1-S Phase Transition by Targeting Bmi-1 in Breast Cancer. The article has since been corrected online.
